# Dilatometric Analysis of the Austenite Decomposition in Undeformed and Deformed Low-Carbon Structural Steel

**DOI:** 10.3390/ma13235443

**Published:** 2020-11-29

**Authors:** Mateusz Morawiec, Adam Skowronek, Mariusz Król, Adam Grajcar

**Affiliations:** Department of Engineering Materials and Biomaterials, Silesian University of Technology, 18A Konarskiego Street, 44-100 Gliwice, Poland; mateusz.morawiec@polsl.pl (M.M.); adam.skowronek@polsl.pl (A.S.); mariusz.krol@polsl.pl (M.K.)

**Keywords:** structural steel, low-carbon steel, dilatometric analysis, phase transformation kinetics, CCT diagram, DCCT diagram

## Abstract

This paper aims to analyze the effect of deformation on the phase transformation kinetics of low-carbon structural steel. The steel used for the investigation was subjected to two different dilatometric analyses using a DIL 805A/D device. The first analysis was to determine the phase transformation kinetics without deformation of austenite before cooling. Then, the analysis under deformation conditions was conducted to investigate the deformation effect on the transformation kinetics. Microscopic studies by light microscopy were performed. The essential part of the research was hardness analysis for different cooling rates and the creation of continuous-cooling-transformation (CCT) and deformation continuous-cooling-transformation (DCCT) diagrams. It was found that the deformation of the samples before cooling increases a diffusion rate in the austenite resulting in the corresponding increase of ferritic, pearlitic, and bainitic start temperatures, as well as shifting the austenite transformation product regions to a longer time. The increase of the transformation area and a decrease in grain size are observed for the deformed samples.

## 1. Introduction

Conventional low-carbon structural steels are still one of the most used steel grades of wide application areas [1,2]. The reason for this is their low price, good weldability, formability, and their lean chemical composition [3,4]. These steels are composed of a ferritic matrix with pearlite, which amount depends on a carbon level. These steels are used for many elements, which are usually produced by different hot-working and cold metal forming methods [5,6]. That is why it is important to comprehend the plastic deformation influence on the phase transformation kinetics of these steels, which selects the best deformation/heat treatment conditions for obtaining the desired microstructure and mechanical properties [7,8,9]. Deformation strongly influences the phase transformation kinetics [10,11], which is caused by the change in a state of microstructure. Undergoing deformation, the dislocation density in austenite increases leading to a higher preferential ferrite/bainite nucleation sites, such as grain boundaries or shear bands [12,13]. On the other side, a thermal cycle (heating and cooling) also influences the phase transformation kinetics. The start and finish transformation temperatures depend on the heating and cooling rates [14,15]. The austenite transformation to ferrite/pearlite/bainite and martensite depends on a cooling rate, which is directly connected to the steel hardenability [16]. The best way to determine the phase transformation kinetics in undeformed and deformed states is a dilatometric analysis [17]. This method uses the principle of linear thermal expansion of a sample during heating and cooling to determine the start and finish temperatures of different phases during heat treatment. The same phenomenon is used during the deformation of the sample. Based on the results from the dilatometric analysis, together with microstructure investigations and hardness measurements, it is possible to determine the phase transformation kinetics and to develop continuous-cooling-transformation (CCT and DCCT) diagrams [18,19]. These diagrams present the areas of transformations taking place at different deformation and heat treatment conditions. A number of works focus on presenting the effect of cooling rate on the kinetics of phase transformations. However, in industrial conditions, the vast majority of this process is associated with the hot-working that takes place during the production and forming processes. Considering the above issues, the following work aims at determining the effect of heat treatment and deformation on the kinetics of phase transformations in low-carbon structural steel.

## 2. Materials and Methods

**Figure 1:**
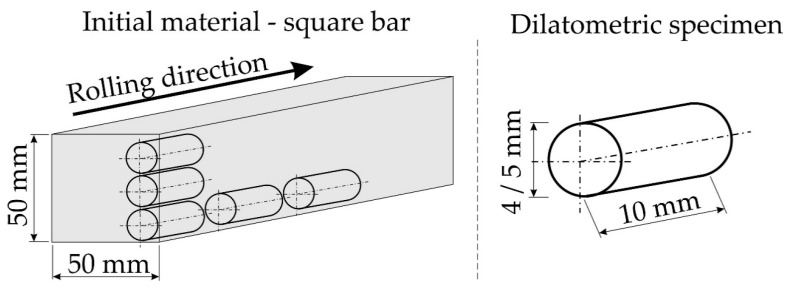
The geometry of the initial material and dilatometric specimens.

The investigated steel is a conventionally used low-carbon s235JR grade structural steel. The mechanical properties of this steel are: Yield point 235 MPa, tensile strength 340 MPa, and the total elongation of 26%. The chemical composition of the investigated steel was 0.2% C, 1.5% Mn, and 0.009 and 0.045% of S and P, respectively. A steel bar with a 50 × 50 mm^2^, square section was used for the investigations (Figure 1). The first step of the analysis was a dilatometric test. The samples of 4 mm and 5 mm in diameter, and 10 mm length were machined. The tests were performed using a BAHR dilatometer 805 A/D (TA Instruments, Wetzlar, Germany) with a vacuum chamber and induction heating. The investigation and analysis of dilatograms and determination of critical temperatures were made according to ASTM A1033-04 [20]. One sample was subjected to very slow heating to 1100 °C at a rate of 0.25 °C/s to determine the austenite start (A_c1_) and finish (A_c3_) temperatures of the steel. For the purpose of CCT and DCCT diagrams, the samples were heated to 1050 °C at a rate of 1 °C/s.

**Figure 2:**
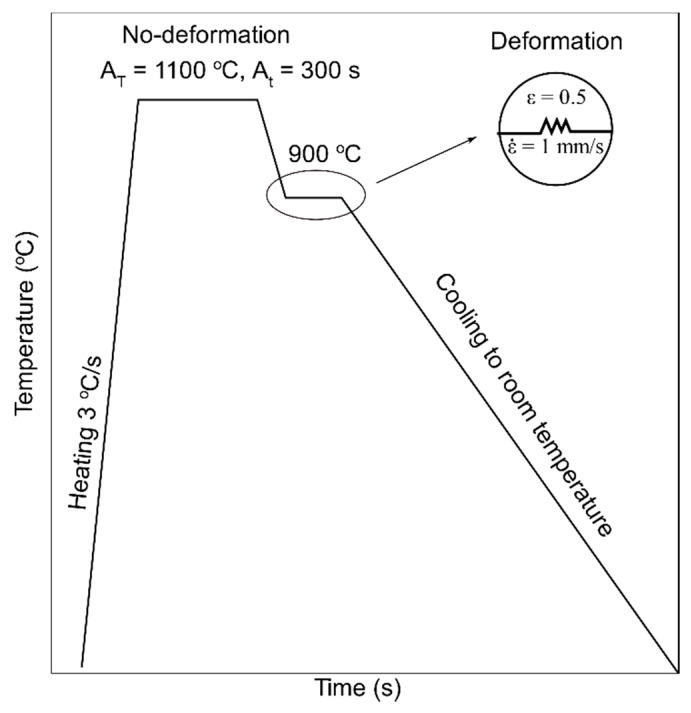
Thermal cycles of dilatometric analysis for non-deformed and deformed austenite; A_T_—austenitizing temperature, A_t_—austenitizing time.

After obtaining the austenitization temperature, the samples were held for 5 min to homogenize the temperature and chemical composition. Next, the samples were cooled to 900 °C at a rate of 4 °C/s, kept for 20 s, and cooled to room temperature at different rates. In the case of deformation, the samples were subjected to 50% deformation at 900 °C at a rate of 1 mm/s, after which they were cooled to room temperature (Figure 2). The heating process was conducted in a vacuum, whereas, the cooling of samples to room temperature was performed using argon. The selected cooling rates are presented in Table 1.

After the dilatometric analysis, the samples were prepared for metallographic investigations using standard metallography procedures [21]. The samples were cut in half (non-deformed samples) and in 1/3 of the length parallel to the deformation direction (the representing deformation region for compressed specimens) and ground using various SiC-based papers of: 220, 500, 800, and 1200 gradation. After grinding, the samples were polished using the diamond paste of 3 and 1 µm and etched in 5% Nital.

The effect of the different heat treatments on the mechanical properties was assessed using hardness tests. They were performed using the Vickers method with a load of 9.81 N (HV1).

## 3. Results and Discussion

### 3.1. Dilatometric Analysis

**Figure 3:**
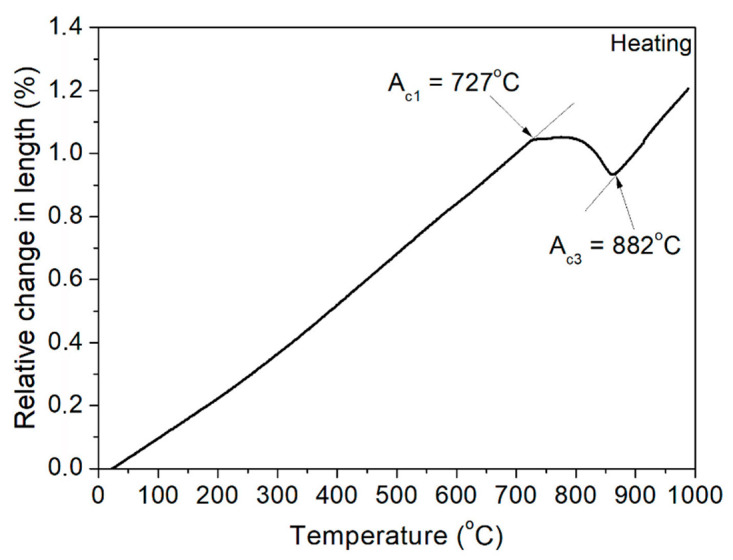
Dilatometric analysis of A_c1_ and A_c3_ temperatures.

**Figure 4:**
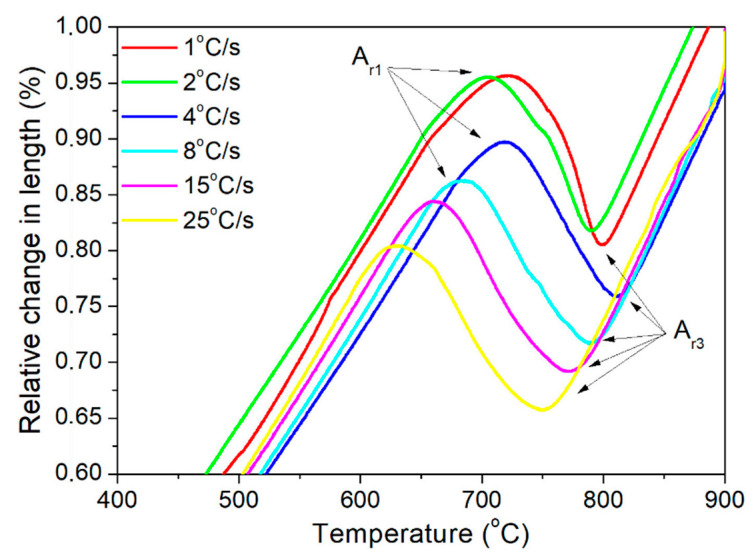
Dilatometric curves of undeformed steel samples at different cooling rates.

The first step of the analysis was to determine the austenite formation start (A_c1_) and finish (A_c3_) temperatures of steel during heating. For this purpose, a sample was heated to 1100 °C at a rate of 4 °C/min to simulate near-equilibrium conditions. The results of this analysis are presented in Figure 3. Based on the dilatometric results, the critical temperatures were determined. The austenite transformation starts at 727 °C and finishes at 882 °C. After determining the critical temperatures, the analysis of the phase transformation kinetics during cooling was performed. Results are presented in Figure 4. It can be noticed that with increasing cooling rate, the start and finish transformation temperatures decrease in undeformed samples. The same results were obtained for deformed conditions. The reason for this is a decreasing diffusion time at faster cooling rates [22,23,24]. When the cooling is faster, it takes a longer time to start the diffusion processes. In this case, for ferrite and pearlite transformations, a longer time is necessary for the transformation to be completed. In the case of deformed samples, one more thing was determined, namely, the phase transformation temperatures are higher compared to the same cooling rate in non-deformed samples. This effect corresponds to the higher dislocation density [25] in the austenite deformed at 900 °C. It leads to a higher number of preferable places for ferrite nucleation and a resulting increase in transformation temperatures [26,27,28]. In industrial applications, understanding this relationship is very important for obtaining proper microstructures.

**Figure 5:**
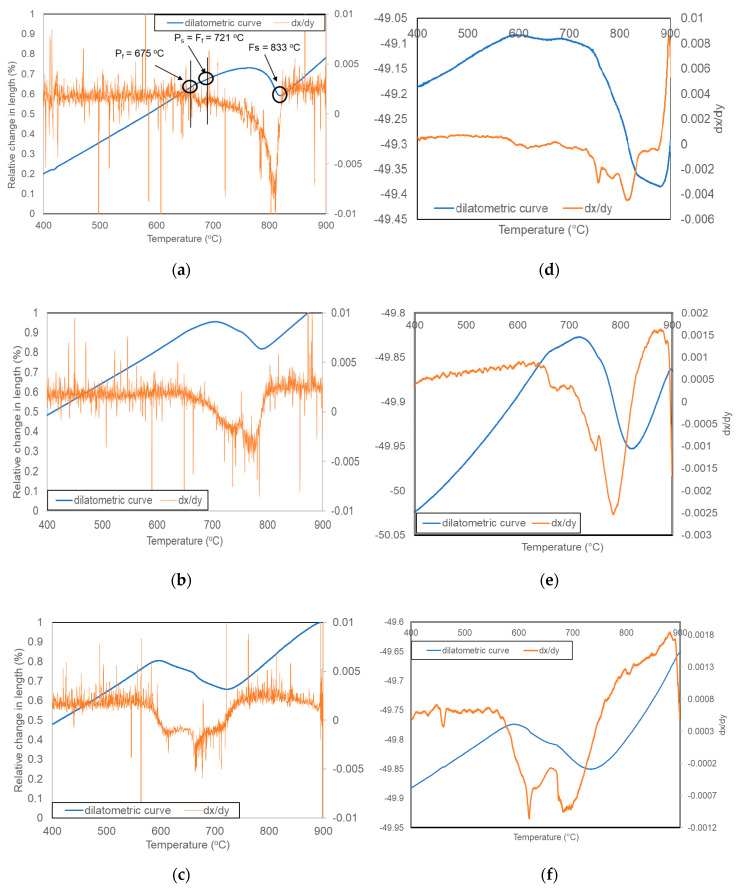
Dilatometric and differential curves of undeformed (**a**–**c**) and deformed (**d**–**f**) samples subjected to different cooling rates: (**a**,**d**): 8 °C/min, (**b**,**e**): 2 °C/s, (**c**,**f**): 25 °C/s.

**Figure 6:**
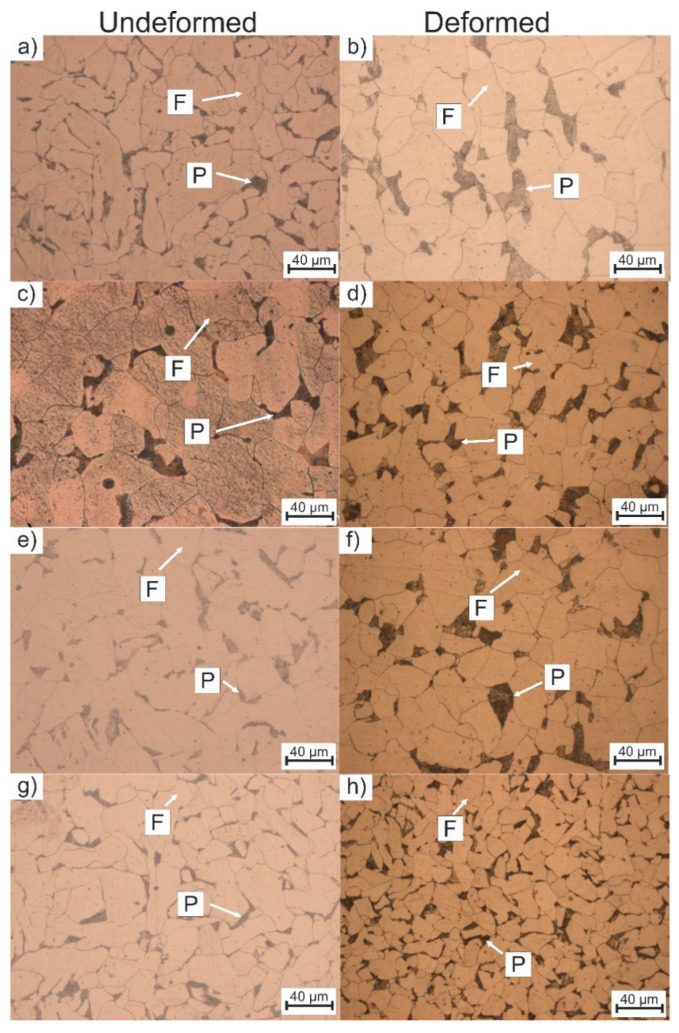
Microstructures of non-deformed and deformed samples at different cooling rates: (**a**,**b**): 0.125 °C/s, (**c**,**d**): 1 °C/s, (**e**,**f**): 2 °C/s, (**g**,**h**): 4 °C/s.

The selected registered dilatometric curves are presented in Figure 5. The results show the dilatometric and differential curves for undeformed and deformed samples cooled to room temperature at a rate of: 8 °C/min (Figure 5a,d), 2 °C/sec (Figure 5b,e), and 25 °C/sec (Figure 5c,f). Figure 6a also shows the way used for the determination of ferritic and pearlitic transformation start and finish temperatures. The pearlitic transformation, in this case is hard to be identified using only dilatometric curves. They only show the start and finish temperatures of the whole transformation, which prevents a good identification of temperatures at which different transformations take place. This is the reason why the differential curve was used.

The first peak on a differential curve corresponds to a ferrite transformation. This phase is created first because of a low carbon concentration in steel, which is too small for pearlite forming in the initial state of transformation. After the first peak, the second small one can be seen, which represents the pearlite transformation. The size of the second peak reflects the amount of pearlite in the microstructure. The shape of the peaks corresponds to the amount of a phase formed during cooling. As the peak is wide, it means that the transformation takes a longer time to be finished. At the same time, an increase in the peak height is correlated with the power of the signal from the transformation. The more phase is formed, the higher peak is observed [29]. For this cooling rate, the microstructure should be composed mainly of ferrite with a small fraction of pearlite. In this case, the ferrite start temperature (Fs) was determined to be 833 °C; for the pearlite, the transformation starts temperature (Ps) was 721 °C, which at the same time was the ferrite finish temperature (Ff). The pearlite transformation is completed (Pf) at 675 °C, indicating in this case, the whole transformation finish temperature.

### 3.2. Microstructure Evolution

**Figure 7:**
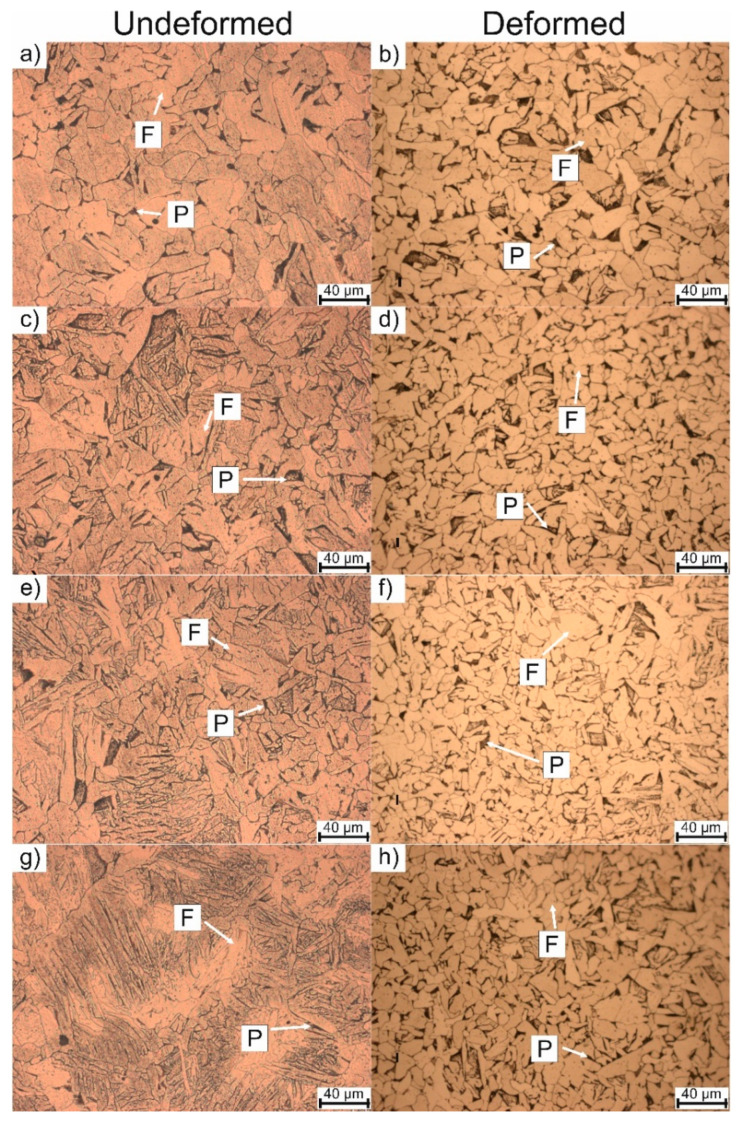
Microstructures of non-deformed and deformed samples at different cooling rates: (**a**,**b**): 8 °C/s, (**c**,**d**): 15 °C/s, (**e**,**f**): 25 °C/s, (**g**,**h**): 50 °C/s.

The microstructure investigations after the dilatometric analysis of non-deformed and deformed samples are presented in Figure 6 and Figure 7. It can be seen that for the non-deformed samples, the microstructure comprises the ferritic matrix with some fraction of pearlite. Starting from the cooling rate of 0.125 °C/s to 4 °C/s the morphology of the ferrite is regular (globular shape) (Figure 6a,c,e,g), but when the cooling rate increases to 15 °C/s and faster rates (Figure 7c,e,g) the ferrite changes its morphology to more lath-like [30]. This kind of morphology is present because the cooling rate is still too low to produce bainite or martensite (low hardenability of the steel) [31], but fast enough to decrease the necessary time for ferrite to form globular grains. This kind of lath-like morphology (Figure 7g) slightly increases the hardness of the ferrite.

In the case of deformation, the samples present globular grains in the whole range of cooling rates. The plastic deformation also reduces the grain size. This phenomenon is described by Inoue et al. [32]. Additionally, when the cooling rate increases to 8 °C/s, a small fraction of globular bainite is present in the microstructure (Figure 7b). The microstructure does not show any type of lath-like morphology compared to the non-deformed samples. The reason for this may be a higher phase transformation start temperature for ferrite, as the dilatometric analysis indicated.

Based on the obtained microstructures, a mean diameter of the grain was calculated according to ASTM E112-113 standard [33]. The results of this analysis are presented in Table 2. According to the results, it can be seen that the increase in a cooling rate leads to a decrease in the grain size diameter. At the same time, the deformation of the material results in further grain refinement of the grains. However, the change of grain size after the deformation is not significant in this steel. Moreover, for the highest cooling rate, the undeformed samples are characterized by lath-like morphology. This makes it impossible to determine the grain size.

### 3.3. Hardness Analysis

**Figure 8:**
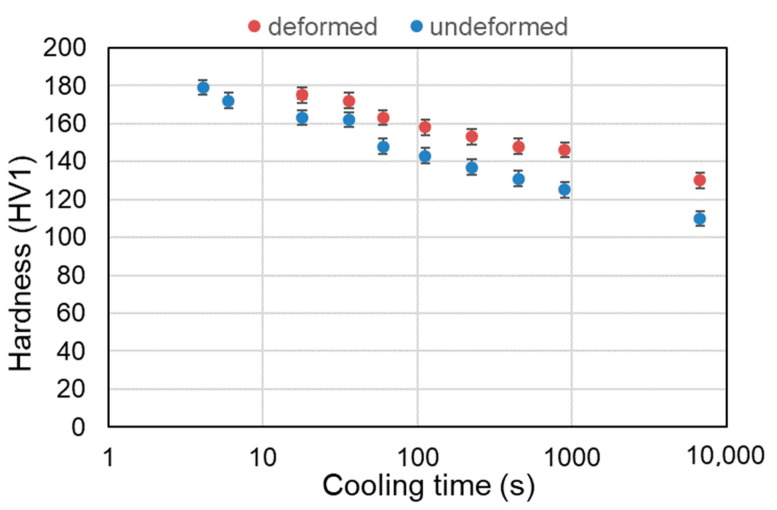
Hardness results for the undeformed and deformed samples cooled at different rates.

The next step of the work was the analysis of the steel hardness after the dilatometric tests. The hardness was measured by the Vickers method. During the test, ten values were registered, and the average was calculated. The results of the hardness measurements are presented in Table 3, and a change in hardness as a function of cooling rate is illustrated in Figure 8.

The hardness changes together with different cooling rates; higher cooling rates applied during the cooling increase the hardness of the material. The reason for this is a smaller grain size of samples cooled at faster rates according to the Hall-Petch relationship [34]. The second effect corresponds to a shorter time (for the faster cooling rate) for the carbon diffusion during cooling, which leads to a higher carbon concentration in the phase. For the higher cooling rates, the hardness increase corresponds to the production of higher strength phases [35] (in this case bainite). The hardness of the material subjected to the deformation is slightly higher compared to non-deformed samples. The reason for this is a stronger grain refinement after deformation, which further increases the strength of the steel.

### 3.4. CCT and DCCT Diagrams

**Figure 9:**
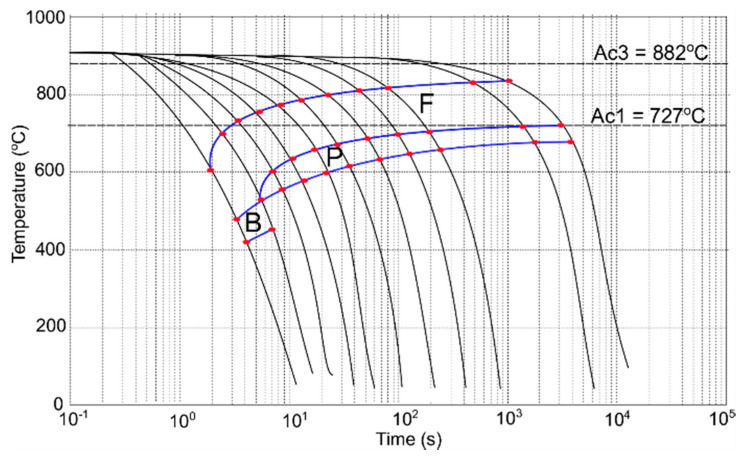
Continuous-cooling-transformation (CCT) diagram of the investigated low-carbon structural steel; blue lines denote the phase transformation zones.

**Figure 10:**
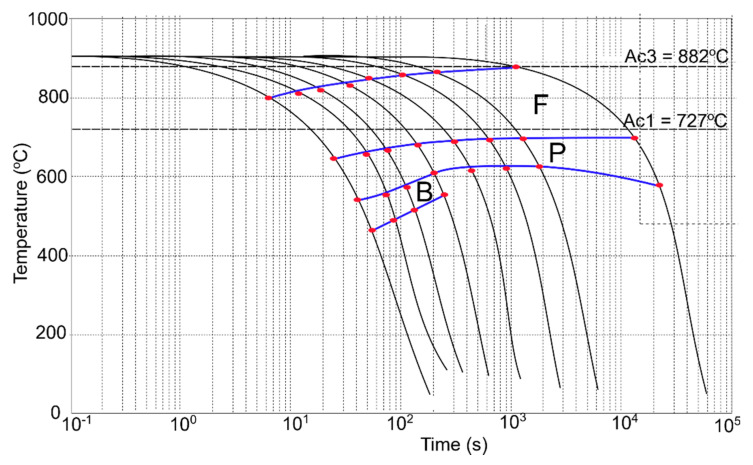
Deformation continuous-cooling-transformation (DCCT) diagram of the investigated low-carbon structural steel; blue lines denote the phase transformation zones.

The final step of the investigation was preparing CCT and DCCT diagrams based on the dilatometric curves, microstructure analysis, and hardness measurement. The results of the mentioned analyses for undeformed and deformed samples are presented in Table 4 and Table 5. Based on the above-mentioned results, both diagrams were created. The corresponding CCT and DCCT diagrams are presented in Figure 9 and Figure 10, respectively. The diagrams are created by connecting the red dots corresponding to the characteristic temperatures listed in Table 4 and Table 5. The color lines connecting the dots (the blue lines in Figure 9 and Figure 10) form the phase transformation zones of the different structural constituents.

Based on presented diagrams, it can be stated that the deformation enhances the diffusion intensity [35]. This phenomenon is revealed as a shift of individual phase regions. After deformation, the ferrite, pearlite, and bainite start temperatures increase to higher values, and the phase areas (ferrite and pearlite) are wider when compared to the undeformed material (Figure 10). Similarly, the phase transformation areas are shifted to lower cooling rates. In the case of bainite, a necessary minimal cooling rate for its formation drops from 150 °C/s to 8 °C/s after deformation. Therefore, for low-carbon structural steels of low hardenability, the deformation before cooling is a good way for increasing their strength by grain refinement and induction of hard phases.

## 4. Conclusions

The following work concerns the effect of heat treatment and hot deformation on the kinetics of phase transformations in low-carbon structural steel.

- The analyzed low-C steel is suitable for the production of ferrite-based microstructures because of its low carbon content and resulting in low hardenability.
- The hot deformation performed before cooling increases the diffusion rate of elements and highly influences the phase transformation kinetics.
- The deformation shifts the phase transformation product regions to higher temperatures and slower cooling rates.
- The deformation causes an increase of strain-induced preferable nucleation places for ferrite, pearlite, and bainite formation, which are diffusional or semi-diffusional (bainite) phase transformations.
- The necessary minimal cooling rate for bainite formation strongly decreases after deformation from 150 to 8 °C/s.
- The plastic deformation substantially enhanced grain refinement in the whole range of applied cooling rates.
- The hardness of the steel increases along with the increasing cooling rate, due to progressive grain refinement and lower transformation start temperatures, also inducing smaller grain sizes.

## Figures and Tables

**Table 1:** Cooling rates selected for the dilatometric analysis.

Sample	Undeformed	Deformed
1	0.125 °C/s	0.125 °C/s
2	1 °C/s	1 °C/s
3	2 °C/s	2 °C/s
4	4 °C/s	4 °C/s
5	8 °C/s	8 °C/s
6	15 °C/s	15 °C/s
7	25 °C/s	25 °C/s
8	50 °C/s	50 °C/s
9	150 °C/s	-
10	220 °C/s	-

**Table 2:** Grain size diameter as a function of the cooling rate for non-deformed and deformed samples.

Cooling Rate, °C/s	0.125	1	2	4	8	15	25	50
ND, µm	53	50	34	34	34	19	15	LM
D, µm	53	49	30	30	30	15	14	13

Note: ND—non-deformed, D—deformed, LM—lath morphology.

**Table 3:** Hardness measurements of non-deformed and deformed samples.

Cooling Rate	Hardness, HV1	
	Non-Deformed	Deformed
8 °C/min	110 ± 3	130 ± 4
1 °C/s	125 ± 3	146 ± 3
2 °C/s	131 ± 3	148 ± 3
4 °C/s	137 ± 3	153 ± 4
8 °C/s	143 ± 4	158 ± 3
15 °C/s	148 ± 3	163 ± 3
25 °C/s	162 ± 3	172 ± 3
50 °C/s	163 ± 4	175 ± 3
150 °C/s	172 ± 3	-
220 °C/s	179 ± 3	-

**Table 4:** Transformation start and finish temperatures are determined based on dilatometric analysis for undeformed samples.

V_cooling_ °C/s	Hardness HV1	Transformation Start and Finish Temperatures					
		B_s_	B_f_	P_s_	P_f_	F_s_	F_f_
220	179	482	415	-	-	605	482
150	172	550	516	-	-	673	550
50	163	-	-	602	550	698	602
25	162	-	-	637	582	748	637
15	148	-	-	650	609	764	650
8	143	-	-	683	618	789	680
4	137	-	-	682	640	797	682
2	131	-	-	692	648	806	692
1	125	-	-	707	653	811	707
0.125	110	-	-	714	672	826	714

Note: B_s_—bainite start temperature, B_f_—bainite finish temperature, P_s_—pearlite start temperature, P_f_—pearlite finish temperature, F_s_—ferrite start temperature, F_f_—ferrite finish temperature.

**Table 5:** Transformation start and finish temperatures are determined based on dilatometric analysis for deformed samples.

V_cooling_ °C/s	Hardness HV1	Transformation Start and Finish Temperatures					
		B_s_	B_f_	P_s_	P_f_	F_s_	F_f_
50	172	541	470	660	541	800	660
25	165	560	485	660	560	810	660
15	163	569	492	668	569	820	668
8	158	626	542	673	626	820	673
4	153	-	-	680	641	857	680
2	148	-	-	682	639	860	682
1	146	-	-	683	642	862	683
0.125	130	-	-	685	576	882	685
